# The levels problem in psychopathology

**DOI:** 10.1017/S0033291719002514

**Published:** 2021-04

**Authors:** Markus I. Eronen

**Affiliations:** Department of Theory and History of Psychology, University of Groningen, Grote Kruisstraat 2/1 9712 TS Groningen, Netherlands

**Keywords:** Integration, levels, pluralism, reduction, scale

## Abstract

Psychiatric disorders are studied at multiple levels, but there is no agreement on how these levels are related to each other, or how they should be understood in the first place. In this paper, I provide an account of levels and their relationships that is suited for psychopathology, drawing from recent debates in philosophy of science. Instead of metaphysical issues, the focus is on delivering an understanding of levels that is relevant and useful for scientific practice. I also defend a pragmatic approach to the question of reduction, arguing that even in-principle reductionists should embrace pluralism in practice. Finally, I discuss the benefits and challenges in integrating explanations and models of different levels.

## Introduction

Psychiatric disorders are studied at multiple levels, the most salient ones being the psychological level and the biological level. At the psychological level, we find things like psychological symptoms, cognitive processes and dysfunctions, and affect states. At the biological level, we find things like brain states, neurons, genes, and so on. The biological level can also be further broken down to more specific levels, such as the level of molecules, cells, and brain circuits.

Although talk of such levels is ubiquitous in the literature, there is no agreement on how they are related to each other, or how they should be understood in the first place (Miller, 2010; Thomas and Sharp, 2019). However, in recent years, there have been important developments in philosophy of science (especially philosophy of biology) concerning levels and cross-level relationships. In this paper, I will go through these developments, focusing on the ones that are most relevant for psychopathology. I will first discuss different notions of levels and highlight some important differences between levels in the biological sciences and levels in psychopathological research (section ‘Levels and their fuzzy boundaries’). Subsequently, I will focus on the relationship between levels, and consider the prospects of explanatory reductionism, where biological levels are preferred over the psychological level (section ‘Explanatory reductionism? A pragmatic approach’). Finally, I will discuss the integration of different explanatory levels, also emphasizing challenges and important topics for future research (‘Explanatory pluralism and integration’).

Even though I will discuss reductionism, I will sidestep traditional philosophical debates on issues such as multiple realizability, supervenience, and causal exclusion. Interesting as they are, these debates have been extensively discussed by others, also in the context of (clinical) psychology (see, e.g. Ross and Spurrett, 2004; Borsboom *et al*., 2019 and the commentaries adjoining both papers). Instead, I will introduce a pragmatic framework for understanding levels, reduction, and integration; ‘pragmatic’ in the sense that the aim is not settle the deep metaphysical questions, but rather to find the conceptual framework that is the most helpful for advancing psychopathological research here and now.

## Levels and their fuzzy boundaries

Talk of levels is very common in science, and it is equally common to leave them undefined and let the reader fill in the details with her intuitions and preconceptions. Fortunately, there has been much progress in recent philosophy of science in explicating and clarifying talk of levels (Craver, 2007, 2015; Wimsatt, 2007; Love, 2012; Potochnik and McGill, 2012; Eronen, 2013, 2015; Bechtel, 2008; Brooks, 2017; DiFrisco, 2017; Brooks and Eronen, 2018). Here I will draw from this literature and go through three different ways of thinking about levels: part-whole levels, levels based on the size scale, and levels based on the time scale. These are certainly not the only ways of construing or defining levels, but as I will argue, they provide a useful basis for understanding levels in psychopathology.^1^^†^

Levels defined in terms of *part-whole* relationships are central in science, especially in biology. One can find part-whole hierarchies almost everywhere in the biological world: a population is composed of organisms, an organism is composed of tissues, and tissues are composed of cells. A cell is composed of cell parts (organelles), cell parts are composed of molecules, and molecules are composed of atoms. This compositional organization can be characterized in terms of part-whole levels: parts are at a lower level, and the wholes that they form are at a higher level.

This basic idea of part-whole levels is intuitive and clear: living things are made up of parts that form wholes, and the wholes can be said to be at a higher level than their parts. However, it is important to keep two things in mind when referring to such levels in models and explanations. First, part-whole hierarchies and the corresponding levels are typically *local*. There is no uniform layer-cake of levels that would apply to the whole of nature; instead, what part-whole levels there are depends on the system, mechanism or phenomenon to be explained, so that even within one organism there will be many different level-hierarchies (Bechtel, 2008; Craver, 2007; Love, 2012; Winther, 2006).^2^ Second, part-whole levels (like most other notions of levels) are always to some degree idealizations. There will always be entities that fall between levels, and there is often no clear way of determining whether two things are at the same level or not (Eronen, 2013, 2015; Eronen and Brooks, 2018).^3^

A different but equally important way of approaching levels in biology is to understand them in terms of *scales* (Noble, 2012; Potochnik and McGill, 2012; Eronen, 2013, 2015; DiFrisco, 2017; see also Rueger and McGivern, 2010). Often the focus of interest is not on compositional relationships (i.e. what are the parts and what are the wholes), but rather in the *scale* of the things and phenomena that are studied. For example, both functional neuroimaging and cellular neuroscience aim at studying the activity of neurons, but at different *spatial scales*: in neuroimaging, at the scale of millions of neurons per voxel, and in cellular neuroscience, at the scale of single neurons. The spatial scale is thus defined in terms of the size of the things studied.

In addition to the spatial scale, another crucially important scale in (neuro)biological research is the *temporal* scale, which is based on the rate at which things interact or processes unfold (Wimsatt, 2007; DiFrisco, 2017). The interactions between neurotransmitters and receptors are extremely fast, whereas the interactions between neurons (action potentials, depolarization, etc.) are slower by several orders of magnitude, and the interactions between brain areas or circuits are again far slower.

Although scale-based levels and part-whole levels may sometimes partially overlap, it is important to keep them distinct (Eronen, 2013, 2015). First, things that are at different scales need not be in a part-whole relationship to one another: for example, the firing of neurons takes place at a faster time scale (and smaller size scale) than digestion, but the neurons that fire need not be part of the digestive system. Second, a single part-whole level may encompass things of very different scales (Eronen, 2013, 2015). For example, the components of a cell include things as different as mitochondria, proteins, and free-floating sodium ions, belonging to very different temporal and spatial scales. A further key difference between scales and part-whole relationships is that whereas part-whole hierarchies are discrete, scales are entirely continuous: it is not necessary to draw a boundary where, say, the scale of molecules ends and the scale of neurons begins.

As this brief overview suggests, much of the levels-talk in (neuro)biology can be understood in terms of either scales or part-whole relationships. However, things get trickier when we turn to the *psychological* level in psychopathology. What is exactly meant with ‘the psychological level’ is rarely if ever explicitly stated, but is typically characterized through examples: things such as affect states (e.g. fear, sadness, happy mood) and psychological symptoms (e.g. rumination, suicidal thoughts, hallucinations) are mentioned as paradigmatic examples. The first complication is that the boundary between the psychological and the biological is often fuzzy. Consider, for example, the symptoms of depression. Is insomnia a psychological or biological symptom? The inability to fall asleep can be seen as a psychological state, but on the other hand, when the body is not in a sleeping state, this is also a thoroughly biological issue with important biological and physiological consequences. Similarly, it is not clear whether we should consider (mild) cognitive impairments as psychological or neurobiological phenomena, or both.

A second problem is that it is not easy to extend the typical biological levels (part-whole levels and scale-based levels) to cover also psychology. Starting with part-whole levels, although such levels can also be discerned among psychological phenomena, they are different from biological part-whole levels. For example, symptoms make up mental disorders: a disorder such as depression is made up of its symptoms, such as sadness and rumination (at least according the view defended by, e.g. Borsboom & Cramer, 2013). This suggests that there is a part-whole relationship between symptoms and the disorder, and in this sense symptoms are at a lower part-whole level than the disorder. However, in contrast to biological part-whole levels of the kind described above, these part-whole levels do not involve clearly identifiable material parts, such as neurons or brain structures. Instead, there are psychological states or constructs (e.g. symptoms) that make up more complex psychological states or constructs (e.g. a disorder). In contrast to biology, such constructs are often defined in an indirect way (e.g. based on operationalizations or indicators) and without a solid theoretical basis.

Similar considerations apply to the size scale. How big is the state of ruminating? It could be argued that the state of ruminating occupies more space than the more fine-grained mental states that it is composed of. However, this sense of the spatial size is rather metaphorical, and very different from the context of biology, where volume or extension of things like cells or organs can be measured. In contrast, the *temporal* scale is can be extended to the context of psychology in a rather straightforward way. Psychological symptoms and states such as contamination fear, rumination, and thinking about dinner unfold over time. Although the exact rate at which they take place may be difficult to estimate or measure, it makes sense to analyze and place them on a temporal scale. Importantly, in many cases the rate will be much slower than for (neuro)biological states or processes. For example, the states of brain circuits and neurons change at the scale of seconds or milliseconds, whereas psychological states are more likely to change over minutes, hours or even days. These differences in time scales could be used to define levels, but as scales are continuous, where the boundary between levels should be drawn may not have a clear answer (see also DiFrisco, 2017).

The upshot is that defining levels in psychopathology is far from straightforward. The exact levels and their significance can vary strongly from context to context. In psychology, it is often unclear how part-whole or scale-based levels should be understood in the first place. This suggests that levels in psychopathology are best seen as heuristic idealizations that are helpful in making rough distinctions, but do not mark deep ontological features of the world (Brooks and Eronen, 2018). Talk of levels is useful for framing the debate on issues such as reduction and pluralism (as in the next sections), but the exact levels themselves should not be given too much emphasis (see also Campbell, 2008; Murphy, 2017).^4^

## Explanatory reductionism? A pragmatic approach

Above I have argued that levels can be defined in various ways and typically have fuzzy boundaries, but can nevertheless be heuristically useful. In this section, I will turn to the question of reduction. Instead of discussing the age-old metaphysical issues related to the mind-body problem, I will focus on the question of how *explanations at different levels* are related to each other. More specifically, I will consider the prospects of *explanatory reductionism* in psychopathology. Such explanatory reductionism comes in different versions. In its most ‘ruthless’ form (Bickle, 2003, 2015), it amounts to claiming that explanations at the psychological level are not even real explanations, but only appear to be so because we do not yet understand the neurobiological mechanisms of the brain. According to a slightly milder version, the psychological level can provide real explanations, but they are less powerful or important than neurobiological explanations, so it is better to direct research efforts to neurobiological levels (Insel, 2010; Insel and Cuthbert, 2015; Gordon, 2016). In both versions, the key idea is that the relationship between the psychological and biological level(s) is unequal and asymmetric in the sense that lower-level (biological) explanations are somehow ‘better’ than higher-level (psychological) ones.

There are several good reasons for thinking this. First of all, there is little doubt that the basis for psychological phenomena, at least in principle, lies in the biological mechanisms of the brain and the body. Mental phenomena, even if they are socially and culturally influenced, are in the end realized in the firing of neurons and physiological processes in the body. This is clearly expressed in the introduction to *The Principles of Neural Science*, one of the standard textbooks on neuroscience: ‘we emphasize … that behavior can be examined in terms of the electrical activity of both individual neurons and systems of nerve cells … we document the central principle that all behavior is an expression of neural activity’ (Kandel *et al*., 2012). If we accept this premise, it seems plausible that the behavior and mental phenomena should also be studied directly at the level of neurons or neural activity, as that is the most fundamental level on which all the rest depends.

This kind of reasoning is also supported by the fact that reductionist research programs that dig deeper and deeper into lower levels have been very successful in other fields such as biology or physics, leading for example to the discovery of the deoxyribonucleic acid (DNA) in biology and the specification of the Standard Model of particle physics. Such research has also led to great improvements in treatments for medical diseases such as cancer or heart disease (Insel and Cuthbert, 2015). In recent decades, there have also been many technological advances in fields that do lower-level research that is relevant to psychopathology, such as neuroimaging, genetics, and cellular and molecular neuroscience, opening up new possibilities for research at the (neuro)biological levels in psychopathology (Walter, 2013). Thus, reductionists argue, the time is ripe to pursue such research in psychiatry as well, with the hope that it will lead to breakthroughs (Insel and Cuthbert, 2015; Gordon, 2016).

Attractive as these arguments are, they do not result in a compelling argument for preferring lower levels in favor of higher ones. Let us start with the success of reductionist research programs. There were indeed great breakthroughs in research at the lower levels in the 20th century, but in recent years and decades the *limits* of reductionism have become increasingly clear, especially in biology and its philosophy. One vivid example is provided by Denis Noble (2006, 2012), who developed the first mathematical model of the heartbeat. He initially set out to find an explanation for the heartbeat at the level of ion channels in cell membranes, looking for a ‘pacemaker’ mechanism. However, it turned out that there is no such mechanism. The ion channels open or close based on the electrical cell potential, and this potential is influenced by the joint activity of ion channels. The rhythm of the heart is a result of these feedback loops, and higher-level properties such as the cell potential and cell structure are essential for understanding the phenomenon. Even though the basis for the heartbeat is known to be in the activity of the ion channels, the best models for predicting, controlling and explaining the heartbeat are found at the higher level of whole cells.

As another example, consider the phenomenon of avian flight. Lower levels such as genetics and molecular biology are part of the story, but at those levels, very few useful regularities can be found. This is due to the complexity of the phenomenon: successfully modeling bird flight requires knowledge from multiple levels and fields, including aerodynamics (the physics of flight), morphology (muscle function, bone structure, etc.), ecology (e.g. the energy costs of flying), and sensory biology (e.g. sensorimotor feedback; Hedenström, 2002; Altshuler *et al*., 2015). Even in physics, explanations at the level of molecules often become intractable, necessitating the use of higher-level models for effectively controlling and predicting the behavior of a system (Bishop, 2008; Green and Batterman, 2017).

The common pattern that emerges from these examples is that explaining or predicting the behavior of complex systems requires higher-level explanations. If we now turn back to mental disorders, what we find is a paradigmatic example of extreme complexity: it is widely thought that the brain is the most complex structure known to science, consisting of ca. 87 00 00 00 000 neurons. Moreover, the dynamics of the interactions between even a small number of neurons can be extremely complex and cannot be read off from just their connections. As Bargmann and Marder (2013) point out, the connectome or ‘wiring diagram’ of the 302 neurons of the roundworm *Caenorhabditis elegans* has been mapped out, but this knowledge is not enough to determine which neurons are central or how the circuit as a whole functions. This is because the function of each neuron depends on the dynamics of the circuit as a whole, and can also change due to chemical neuromodulators (Bargmann and Marder, 2013). These challenges are even more serious when it comes to the human brain, where one neuron can receive input from up to hundreds of thousands of other neurons (Yuste, 2015).

Similar issues related to complexity hinder the search for genetic explanations of mental disorders. In spite of massive efforts, no clinically useful associations have been discovered between specific genes and psychiatric disorders (Munafo *et al*., 2014). The recently developed genome-wide association study techniques allow for investigating the DNA of thousands of participants and then looking for associations between specific variations in the DNA and phenotypes. Such studies have revealed only tiny correlations between mental disorders and specific bits of DNA (or polygenic scores involving several DNA loci; Chabris *et al*., 2015; Turkheimer, 2016). One plausible explanation for this is that mental disorders are simply too complex as phenotypes to yield ‘strong’ genetic explanations in the sense of specific and identifiable genetic mechanisms that are causally related to the disorder (Turkheimer, 2016). The causal chain from a strand of DNA to a disorder such as a depression is very long and convoluted (Chabris *et al*., 2015), and unlikely to be clinically relevant.

In this light, it is perhaps not surprising that reductionistic research programs in psychiatry that target lower biological levels have led to few clinically useful insights in recent decades (Hyman, 2010; Krystal and State, 2014; Frisch, 2016; Borsboom *et al*., 2019). In spite of high hopes, no reliable biomarkers or genetic explanations for mental disorders have been discovered (Kalia and Silva, 2015).^5^ On the other hand, at the psychological levels, the track record is arguably better. To mention just a few examples, research into the cognitive processes underlying obsessive-compulsive disorder has shed light on different subtypes and the etiology of the disorder (Hezel and McNally, 2016), and recent studies have suggested ‘critical slowing down’ at the level of psychological symptoms as a possible early warning signal for depression (van de Leemput *et al*., 2014). It can also be argued that what we are in the end interested in when treating patients is exactly the psychological level, namely the alleviation of psychological symptoms (Miller, 2010; Borsboom *et al*., 2019). If an individual has no psychological symptoms of depression, but a ‘depression circuit’ in the brain (Insel, 2010), it is hard to justify treating that individual as having a mental disorder.

A closely related point is that even if researchers were to succeed in discovering the brain mechanism(s) or genes that underlie a certain mental disorder, this would not mean that psychological explanations regarding that disorder would become worthless or obsolete. For example, we know that smoking is a cause for lung cancer because several independent sources of evidence indicate that the amount that people smoke makes a difference for the occurrence of lung cancer, and intervening on the smoking habits in a population is a way of reducing the occurrence of lung cancer (Woodward, 2003). Even if we were to have figured out the exact molecular mechanisms of how cigarette smoke results in cancerous cell growth, it would nevertheless remain a causal fact that smoking causes lung cancer. Although the biological knowledge might lead to new treatment options, the most effective way of reducing the occurrence of lung cancer would still be intervening on the smoking habits of people, which would prevent them from developing lung cancer in the first place. Similarly, even if we were to be able to figure out the brain mechanisms underlying mental disorders, the (causal) regularities at higher levels that have proven to be useful for predicting, explaining, and treating mental disorders would not lose their importance (see also Eronen, 2010, 2012, 2017; Woodward, 2015).

The main lesson to draw from the examples and arguments in this section is this: even if we assume that mental disorders are in some sense brain disorders, it does not follow that research should be targeted towards lower levels. The central aim of psychopathological research is to find better ways of treating and preventing mental disorders. As mental disorders are extremely complex systems, this is likely to require higher-level models and generalizations. Based on the track record, psychological levels are probably such higher levels where predictive and useful models and generalizations can be found. As Herbert Simon pointed out more than 50 years ago, ‘in the face of complexity, an in-principle reductionist may be at the same time a pragmatic holist’ (Simon, 1962, 86). In order to make progress in understanding and treating psychiatric disorders, research should be pursued at multiple levels, drawing on many different disciplines and approaches. In the next section, I turn to the challenges in integrating these different approaches and levels.

## Explanatory pluralism and integration

Above I have argued that, from a pragmatic point of view, psychopathological research should embrace research at multiple levels. In other words, if the aim is to make progress in treating and understanding mental disorder, *explanatory pluralism* is preferable to explanatory reductionism. Explanatory pluralism in psychopathology has been defended by many authors (e.g. Kendler, 2005; Miller, 2010; Borsboom *et al*., 2019), and it is also inherent in the biopsychosocial model that has been influential in psychiatry at least since the 1970s and is still widely taught to students (Pilgrim, 2002; Frisch, 2016).

However, the basic idea that explanations need to be looked for at multiple levels is compatible with many different scenarios of how such multilevel research will actually play out (Marchionni, 2008; Sullivan, 2014, 2017; Gijsbers, 2016; Love, 2017). Most importantly, pluralism can lead to (1) a patchwork of explanations at different levels, or (2) integration of explanations at different levels. In the first scenario, the different perspectives or different levels that are needed for fully explaining psychopathology cannot be combined to one grand multilevel explanation, but are somehow incompatible or incongruent. In the second scenario, the different perspectives and different levels are compatible and complement each other in such a way that they can be combined to one grand harmonious multilevel explanation. These two scenarios should be seen as the extreme ends of a continuum, as the success of integration is not a yes-or-no thing but a matter of degree.

Most philosophers, researchers, and clinicians would probably agree that something like the integrative scenario is the more preferable and attractive alternative. Many authors have also explicitly advocated integrative pluralism for psychopathology (e.g. Kendler, 2005; Mitchell, 2008; Miller, 2010). However, what has received less attention is how integration would work in practice, and what are the challenges and hurdles that hold back integration. In this section, I will focus on these questions, drawing from recent philosophy of biology where these issues have been more extensively discussed.

First of all, an important feature of integration that is often forgotten in theoretical discussions, also in psychopathology, is that it is *case-specific*. Philosophers and researchers often search for a general answer to the question of reduction and integration, arguing for example that mental disorders can be explained based on brain circuits, or that psychological explanations are always indispensable. In contrast, the degree to which multiple disciplines and levels are needed, and the degree to which the different perspectives can be integrated, can both vary from disorder to disorder (or even from symptom to symptom). The phenomenon that is studied or the problem that needs to be solved (the ‘problem agenda’, Love, 2008) determines what fields and what levels are needed (Love, 2008; Brigandt, 2010). For example, it is plausible that there will be satisfactory low-level biological explanations for disorders such as dementia, but that such explanations will be insufficient for depression or posttraumatic stress disorder (PTSD). Similarly, the integration of explanations or models of different levels might work out well and lead to new insights in one context, but face profound obstacles in another context (see below). In other words, accounts of pluralism and integration in psychopathology should not be overgeneralized, but should be case-specific and sensitive to the scientific details of each case.

Another crucial point is that integration should be seen as an active and dynamic process, and not just as the final goal or end result of pursuing research at different levels. As O'Malley and Soyer (2012) show in the context of systems biology, integration has often led to new insights, or even to the emergence of new and flourishing research fields. They also emphasize that integration does not occur just by combining explanations or theories of different levels, but often involves importing and translating data and models of one discipline to another. For example, in the recently emerged field of evolutionary systems biology, insights and data from evolutionary biology are imported into the cellular and molecular models of systems biology (O'Malley and Soyer, 2012). One recent example of this in psychopathology is the Ising model. This model represents a network of binary variables that interact with their neighbors, and was originally introduced in the 1920s to model the behavior of magnetic particles. However, it turns out that the same model can also be used to describe neural networks (Yuste, 2015), and more recently, the Ising model has been shown to be mathematically equivalent to Item Response Theory models and binary symptom network models in psychology (Van Borkulo *et al*., 2014; Kruis and Maris, 2016; Marsman *et al*., 2018). The fact that integrative multilevel research has been so fruitful in other fields should provide a strong incentive for pursuing such research in psychopathology as well.

However, although it is clear that there is much to be gained from integrating methods, data, and perspectives of different levels, there are several obstacles to such integration in psychopathology. First of all, in the biological sciences integration often occurs through the elaboration of multilevel mechanisms through constraints (Bechtel and Richardson, 1993; Craver and Darden, 2013). The idea is that different fields impose different constraints on what the explanatory mechanism for a phenomenon could be, and in this way the space of possible mechanisms is narrowed down. Often researchers start with a sketch of a mechanism, and as more evidence is gathered, this sketch can be refined and black boxes are filled in. This can involve many different disciplines and perspectives. For example, the discovery and refinement of the model of protein synthesis in the 1950 and 1960s involved integrating knowledge from biochemistry (e.g. chemical reactions involving amino acids) and molecular biology, resulting in constraints on how the mechanism of protein synthesis could look like. Eventually these constraints and inputs from multiple fields resulted in the DNA–RNA theory of protein synthesis.

One obstacle to this kind of integration is the incommensurability of levels discussed in section 2. The part-whole hierarchies in psychology are different from the (mechanistic) part-whole hierarchies in biology, and it is not clear how the two can be integrated. Thus, the mechanistic picture needs to be complemented with an account of how psychological states can be integrated into mechanistic explanations. So far, this has been only done in the context of computational or functional states in psychology (e.g. Piccinini and Craver, 2011; Thomas and Sharp, 2019), but it is not clear how this kind of integration would work for phenomena such as affect states, beliefs, and symptoms. Such integration is also challenged by the fact that psychological processes often unfold and interact at different time scales than biological processes (section 2). Integrating models and explanations that pertain to different time scales are not impossible, but currently there is little understanding on how it should be done.

A more general problem for integration in psychopathology is *descriptive complexity*: different conceptual frameworks often do not carve phenomena in the same way, but result in mismatching and conflicting categorizations (Wimsatt, 1972; Sullivan, 2014, 2017; Tabb and Schaffner, 2017). This can be vividly seen in schizophrenia research. As Sullivan (2014) points out, the cognitive deficits that are important to schizophrenia are studied both in cognitive neuroscience and in cognitive neurobiology, but from different perspectives. In cognitive neuroscience, the aim is to probe specific cognitive functions and to localize them in the brain with neuroimaging techniques. In cognitive neurobiology, the cognitive deficits underlying schizophrenia are studied through animal models (e.g. rats). In such experiments, the aim is to discover differences in the behavior of rats, which are taken to indicate a cognitive deficit, but no mapping to specific human cognitive functions is made. Thus, even though the same phenomenon is nominally being targeted, it is conceptualized in different ways. More generally, Tabb and Schaffner (2017) point out that different state-of-the-art models of schizophrenia that focus on different levels do not even agree on what are the defining features or key symptoms of schizophrenia.

Issues like this abound in psychopathology. For example, fear extinction is studied in neurobiology with rodents based on freezing behavior after a foot shock. In humans, fear extinction is measured with more complex stimuli, and typically with skin conductance responses as the dependent variable (Lonsdorf *et al*., 2017). Recently doubts have been raised regarding this translation, as it is far from clear that the setups are measuring the same phenomena (Lonsdorf *et al*., 2019; see also Glas, 2004; Khalidi, 2005). In psychopathology, descriptive complexity seems to be the rule rather than the exception.

However, this does not mean that there is no hope for integration. In biology, there are many success stories of integrating fields that seem to conceptualize phenomena in different ways (e.g. in the context of systems biology mentioned above; O'Malley and Soyer, 2012). Also in psychopathology, concentrated interdisciplinary efforts, involving both scientists from different fields and philosophers of science, can help to aligning concepts and models of different fields in the context of a specific problem or phenomenon (see also Love, 2008; Sullivan, 2017; Laplane *et al*., 2019).

## Conclusions

In this article, I first pointed out that levels are difficult to define and often have fuzzy boundaries, and that level-hierarchies that make sense in biology (e.g. mechanistic part-whole levels) are not easily extended to cover psychopathology. Levels should not be taken too seriously, but should be seen just as heuristic idealizations. I have also argued that although explanatory reductionism may be appealing in psychopathology, from a pragmatic point of view even an in-principle reductionist should acknowledge the need of pursuing research at higher levels, including the psychological level. Finally, I argued that the integration of explanations and models of different levels can be crucial for making progress in psychopathology, but many key questions are still open: how to integrate psychological states to mechanistic explanations? How to resolve the mismatches between conceptualizations of the same phenomenon at different levels? Studying integration and its challenges is therefore an important topic for further research in psychopathology. In conclusion, there are many unsolved ‘levels problems’ in psychopathology, but studying them in a collaborative way, bringing together philosophy of science and clinical research, can lead to new insights and research avenues.

## Footnotes

† The notes appear after the main text.

## Notes

1 In psychology and cognitive science, Marr's (1982) three levels of analysis (the computational, the algorithmic/representational and the implementation level) are still often mentioned. Although not sufficient for understanding the levels in psychopathological research (Murphy 2017), Marr's levels can still be useful as a heuristic guideline when taking a computational or information-processing perspective to psychopathology (Bechtel & Shagrir 2015; Hardcastle & Hardcastle 2015). They are not in conflict with scale-based or part-whole levels, but should be seen as a further complementary notion of levels.

2 The part-whole relationship itself can also be defined in different ways. For the purposes of this paper, this relationship is best understood as ‘mechanistic composition’ (Craver, 2007; Craver and Bechtel 2007): The wholes are mechanisms performing a certain function, and the parts are components that are relevant for the functioning of the mechanism.

3 For more on complications in defining part-whole levels, see Eronen (2013; 2015).

4 For more detailed discussions of levels, see Bechtel (2017), Craver and Bechtel (2007), Eronen (2013) and Noble (2012).

5 One exception is narcolepsy, which is more a neurological rather than a psychiatric disorder, but is nevertheless included in DSM-5. I thank an anonymous reviewer for pointing this out.
